# Enhancing Mental Health and Cognitive Functioning in Victims of Violence: Cognitive Behavioral Therapy for Sleep Disorders Among Journalists, Human Rights Defenders, and Relatives of Disappeared Persons in Mexico City

**DOI:** 10.3390/bs15040530

**Published:** 2025-04-15

**Authors:** Araceli Martínez-Moreno, Guadalupe Terán-Pérez, Yoaly Arana-Lechuga, Javier Velázquez-Moctezuma, Oscar Sánchez-Escandón, Daniela Guarneros-Roniger, Roberto E. Mercadillo

**Affiliations:** 1 Neurological and Sleep Center, Benito Juárez, Mexico City 03020, Mexico; aramtzmoreno@gmail.com (A.M.-M.); gjovannat@gmail.com (G.T.-P.); yoalysoph@hotmail.com (Y.A.-L.); jvelazquezmoctezuma@gmail.com (J.V.-M.); oscarsanchezescandon05@gmail.com (O.S.-E.); 2 Area of Neurosciences, Biology of Reproduction Department, Universidad Autónoma Metropolitana, Iztapalapa, Mexico City 09340, Mexico; 3 Sleep Disorders Clinic, Universidad Autónoma Metropolitana, Iztapalapa, Mexico City 09340, Mexico; 4 Neurophysiology Service, ABC Hospital, Mexico City 05330, Mexico; 5 Comprehensive Neuropsychology Care Center, Mexico City 04100, Mexico; danielaguarneros@gmail.com; 6 Consejo Nacional de Humanidades, Ciencias y Tecnologías, CONAHCYT, Benito Juárez, Mexico City 03940, Mexico

**Keywords:** violence, sleep, cognitive behavioral therapy, post-traumatic stress disorder, journalists, human rights defenders, disappeared persons

## Abstract

In Mexico, pervasive violence, forced disappearances, and homicides have deeply impacted certain groups, particularly journalists, activists, and human rights defenders, who are at high risk of victimization. While these groups receive state support for physical and legal safety, mental health and sleep-focused interventions remain insufficient. Collaborating with a Mexico City-based institution supporting human rights defenders and journalists, we conducted a psychometric assessment of 47 individuals affected by violence. Results showed that 80% exhibited symptoms of post-traumatic stress disorder, 25.5% showed depression, and 57.4% displayed anxiety; 95.7% reported poor sleep quality based on the Pittsburgh Sleep Quality Index. In a second phase, neuropsychological tests and polysomnographic recordings identified cognitive impairments in attention, memory, and decision-making in some participants, along with sleep disorders such as insomnia, primary snoring, obstructive sleep apnea, and bruxism. A third phase introduced Cognitive Behavioral Therapy for insomnia, nightmares, and circadian rhythm issues. Results showed improvements in sleep quality, total sleep time, and a reduction in depression, anxiety, and post-traumatic stress disorder symptoms. This approach suggests that treating sleep issues in high-risk populations can improve mental health.

## 1. Introduction

Mexico’s “war on drugs” represents one of the most violent periods in the nation’s recent history. According to the National Search Commission (2020), over 60,000 individuals went missing or were forcibly disappeared between 2006 and 2019. Additionally, the National Human Rights Commission (2020) documented 153 cases of journalist homicides from 2000 to 2019, along with 49 homicides of human rights defenders between 2006 and 2019. Exposure to such violence, life-threatening incidents, or the loss of loved ones is closely associated with mental health issues, including depression, anxiety, and post-traumatic stress disorder (PTSD), as observed among victims of various forms of violence (Flores Martínez & Atuesta, 2018). These effects extend to journalists in Mexico (Feinstein, 2012) and other nations (Feinstein et al., 2018), impacting emotional regulation, empathy (Ribero-Marulanda & Novoa-Gómez, 2019), and sleep (Gallegos et al., 2019). Sleep disturbances emerge as a primary concern due to their pivotal role in emotional processing. Disruptions in normal sleep patterns, such as sleep restriction, can impair emotional regulation and social perception of cues related to the self and others (Dorrian et al., 2019). Moreover, PTSD frequently coexists with sleep disturbances such as poor sleep quality, insomnia, or nightmares (Miller et al., 2017).

The relationship between sleep disturbance and cognitive decline is well-documented. Inadequate sleep leads to errors in judgment, impulsivity, and diminished threat recognition, empathy, and emotional regulation. Cognitive functions such as attention, memory, and decision-making also suffer (Koren et al., 2002; Maher et al., 2006; Nappi et al., 2011; Drummond et al., 2006; Sewell et al., 2021). Furthermore, studies indicate that PTSD patients exhibit alterations in neural pathways involving emotion regulation and cognitive functions, implicating regions like the amygdala, hippocampus, and prefrontal cortex (Rabellino et al., 2016; Hughes & Shin, 2011).

Integral treatment for mental disorders, particularly PTSD, is essential, including addressing sleep disorders to prevent exacerbation of daytime symptoms and to facilitate physical and cognitive restoration during sleep (Germain, 2013; Koren et al., 2002; Maher et al., 2006; Nappi et al., 2011; Drummond et al., 2006). Cognitive Behavioral Therapy (CBT) for sleep disorders, comprising educational, sleep hygiene, behavioral, and cognitive components, emerges as the first-line treatment for insomnia as recommended by American and European guidelines and the American Academy of Sleep Medicine (Edinger & Carney, 2014; Manber & Carney, 2015; Wilson et al., 2010).

In response to public forums held in the Mexican Senate in 2016 and 2017, there is a growing demand for mental health support, particularly among journalists, human rights defenders, and families of disappeared individuals, given the pervasive violence in the country (Enciso, 2017; Mercadillo & Enciso, 2018). While institutions have been established by the Mexican government, efforts predominantly focus on providing security, legal aid, and economic support, overlooking mental health, sleep quality, and emotional well-being. Addressing this challenge necessitates that assistance be provided to those continually subjected to re-victimization, persecution, and social stigmas associated with mental illness and violence (Echeburúa et al., 2016). Moreover, there is a scarcity of descriptive, controlled studies guiding interventions, particularly concerning the mental health of journalists and human rights defenders or families of disappeared individuals, with minimal attention given to sleep or emotional regulation.

In summary, the scientific literature has demonstrated that disruptions in sleep patterns are a prominent feature of PTSD symptomatology, and are frequently comorbid with certain neuropsychiatric disorders, particularly depression and anxiety. Poor sleep quality has also been reported to negatively impact cognitive performance, emotional regulation, and empathy. However, there is a notable scarcity of studies examining the relationships between sleep disturbances, mental health, cognition, and emotional skills. Understanding these connections is essential for designing interventions in contexts of violence, the effects of which often encompass depression, anxiety, PTSD, and sleep disturbances—factors that are rarely addressed in research on violence and mental health. Table 1 provides a concise summary of the strengths and limitations associated with the referenced studies.

This study aims to assess sleep disturbances linked to post-traumatic stress disorder (PTSD), anxiety, depression, emotional difficulties and neurocognitive functions among journalists, human rights defenders, and families of disappeared individuals who have endured violence and are in need of assistance. Given the comorbidity between sleep disorders and neuropsychiatric disorders, and considering the history of violence experienced by this population, in this exploratory assessment, we expect to observe a clinical picture characterized by symptoms of PTSD, sleep disorders, difficulties in emotional regulation, and neurocognitive deficits.

Based on the results obtained from the clinical evaluation, we propose a group and individual intervention grounded in Cognitive Behavioral Therapy for sleep disorders. We anticipate that the intervention will not only ameliorate sleep disorders, but also reduce symptoms associated with PTSD, depression, and anxiety, and reverse cognitive impairment.

## 2. General Method

### 2.1. Overview

Our research comprises three sequential studies. The first aims to diagnose the mental health status and assess the incidence of sleep disorders among a sample consisting of journalists, human rights defenders, and/or their relatives who are beneficiaries of the Comprehensive Mechanism for the Protection of Human Rights Defenders and Journalists in Mexico City. This diagnostic evaluation involves identifying psychiatric disorders, sleep disturbances, and social and emotional regulation skills influencing interpersonal relationships.

In the Study 2, neuropsychological and psychiatric profiles are conducted alongside polysomnographic recordings to identify potential neurocognitive alterations, psychiatric symptoms, and sleep patterns requiring attention.

Subsequently, a Cognitive Behavioral Therapy-based intervention for sleep is developed and implemented in the Study 3. The effects of this intervention on sleep quality are assessed using psychometric instruments administered at the intervention’s conclusion, supplemented by testimonials gathered throughout its duration.

### 2.2. Protocol

Recruitment of participants, psychometric, psychiatric, and neuropsychological evaluations, as well as polysomnographic recordings and therapeutic interventions, were conducted at the facilities of two government institutions under the protection and security of the corresponding authorities, under the Mechanism for the Comprehensive Protection of Human Rights Defenders and Journalists of Mexico City, and the Comprehensive Human Rights System of Mexico City. The Institutional Review Board from the Universidad Autónoma Metropolitana-Unidad Iztapalapa approved the described protocol, which adhered to the Ethical Principles of Psychologists and Code of Conduct of the American Psychological Association, as well as the Helsinki Declaration. Each participant voluntarily accepted participation and signed a printed consent form after the procedure and objectives of the evaluation had been explained. All personal information and the identities of the participants were protected in compliance with the Law of Transparency, Access to Public Information, and Accountability of Mexico City, and the Law of Protection of Personal Data in Possession of Obligatory Subjects in Mexico City.

## 3. Study 1: Mental Health and Sleep Diagnosis

### 3.1. Participants

Forty-seven people residing in Mexico City (Mean age = 40.17 ± 12.03, Range = 21–64) who had experienced serious violent events or life-threatening situations within the past three years, resulting in various outcomes, such as bereavement, displacement, unemployment, disruptions in work and routine, relocation to new countries or cultural environments, or family separation, were enrolled in this study (see Table 2).

### 3.2. Procedure

Journalists, human rights defenders, relatives of disappeared individuals, or victims of violence in Mexico were recruited with the assistance of the following two local government institutions in Mexico City dedicated to providing services and support to victims of violence: the Mechanism for the Comprehensive Protection of Human Rights Defenders and Journalists of Mexico City and the Comprehensive Human Rights System of Mexico City. The research team conducted six workshops, each lasting approximately two hours, to educate participants on the importance of mental health and sleep in human life. At the conclusion of each workshop, attendees were invited to participate in a study to assess their mental health, sleep quality, and emotional regulation.

Participants were selected based on the following criteria: (1) Ages between 20 and 65 years old. (2) Individuals who had experienced violence within the preceding three months to three years. (3) If receiving pharmacological treatment, not having changed medications for at least three months prior to the interview. Exclusion criteria included the following: (1) Neurological or psychiatric disorders impairing verbal comprehension and judgment (e.g., dementia or psychosis), and (2) current substance abuse. Both aspects were assessed and diagnosed through clinical interviews, including the MINI-International Neuropsychiatric Interview, conducted by an expert neurologist and psychiatrist who are members of our research team.

Groups of 5 to 8 participants were administered a booklet containing sociodemographic information (gender, education level, marital status, employment) and seven psychometric instruments, which they completed in approximately 60 min. The sessions were always conducted under the supervision of research team members in rooms with suitable lighting and minimal noise.

### 3.3. Psychometric Instruments

The Posttraumatic Stress Disorder Symptom Severity Scale-Revised (EGS-R) (Echeburúa et al., 2016) was used. It consists of 21 items rated on a 0–3 Likert scale to evaluate the global severity of post-traumatic stress symptoms based on the DSM-5: re-experiencing, avoidance behaviors, negative alterations in mood and cognition, and hyperarousal. Cronbach’s alpha for these data was 0.90. The instrument includes three semi-structured items to identify the type (direct/indirect/both) and frequency (once/repeated) of exposure to a traumatic event, and information about previously received treatment (psychological/pharmacological/both). The results are interpreted based on the established cut-off points for the global scale and its subscales. Overall score, cut point = 20; re-experiencing, cut point = 4; avoidance, cut point = 4; hyper-arousal, cut point = 6; cognitive alteration, cut point = 6.

The Beck Depression Inventory II (BDI-II) (Beck et al., 2006) consists of 21 items rated on a 0–3 Likert scale to evaluate depression. Cronbach’s alpha for these data was 0.86. The results are interpreted based on the score: Minimum = 0–13; Mild = 14–19; Moderate = 20–28; Severe = 29–63.

The Beck Anxiety Inventory II (BAI-II) (Beck et al., 1988) consists of 21 items rated on a 0–3 Likert scale to evaluate anxiety. Cronbach’s alpha for these data was 0.88. The results are interpreted based on the score: Minimum = 0–7; Mild = 8–15; Moderate = 16–25; Severe = 26–63.

The Pittsburgh Sleep Quality Index (PSQI) (Jiménez-Genchi et al., 2008) consists of 19 items rated on a 0–3 Likert scale to identify sleep quality for one month before the evaluation, and the presence of sleep disturbances, such as insomnia, obstructive apnea, or nightmares. Cronbach’s alpha for these data was 0.77. The results are interpreted based on the cut point for global scale = 5.

The Sleep Hygiene Practices Scale (SHPS) (Zarcone, 2000) consists of 30 items rated on a 0–5 Likert scale to identify sleep practices (e.g., sleeping and waking at regular hours) for one month before the evaluation. Cronbach’s alpha for these data was 0.76. The results are interpreted based on the score: Poor sleep hygiene = 0–30; Bad sleep hygiene = 31–60; Regular sleep hygiene = 61–90; Good sleep hygiene = 91–120, Excellent sleep hygiene = 121–150.

The Emotion Regulation Questionnaire (ERQ) (Rodríguez-Carvajal et al., 2006) consists of 10 items rated on a 1–7 Likert scale that measure two factors indicating emotion regulation strategies: Cognitive Reappraisal (CR; focused on re-signifying previous events affecting emotional experiences) and Expressive Suppression (ES; focused on the behavioral responses implying the modulation or suppression of emotional expressions). Cronbach’s alpha for these data were as follows: CR = 0.72 and ES = 0.82.

The Interpersonal Reactivity Index (IRI) (Ahuatzin et al., 2019) consists of 28 items rated on a 1–5 Likert scale to evaluate empathic attitudes and dispositions divided into four factors: Perspective Taking (PT; adopting the other’s point of view), Fantasy (FS; empathy for fictional characters), Empathic Concern (EC; feeling compassion for the situations of others), and Personal Distress (PD; automatic contagion of another’s anguish). Cronbach’s alpha for these data were as follows: PT = 0.72; FS = 0.72; EC = 0.62; PD = 0.75.

### 3.4. Data Analysis

A data base was built with the SPSS 20.0 software using the scores given for each instrument to analyze their central descriptors. The data base was examined to verify that all items had been answered and that no missing values were present. Any missing value was detected. Shapiro–Wilk tests were performed to verify the normal distribution for each instrument; a value *<* 0.05 was observed only for the PSQI indicating a non-normal distribution. To verify the previously reported relationships between sleep disturbances, anxiety, depression and post-traumatic stress, as well as to explore the relationships between the latter aspects and emotional abilities, Pearson quotients were executed to correlate the values between the instruments, except for the correlations of PSQI, which were executed by the Spearman quotient since a non-normal distribution was observed for this last instrument.

The ERQ and IRI instruments measure emotional qualities which do not provide cut-points to diagnose disturbances; therefore, Student T-tests were performed to compare our sample values with those previously reported in other Mexican samples not exposed to violence.

### 3.5. Results and Discussion

According to the Posttraumatic Stress Disorder Symptom Severity Scale-Revised, participants reported they have experienced indirect violence (*n* = 22) (e.g., witnessing violent events particularly related to close friends or family), direct violence (*n* = 19) or both (*n* = 6), and that they have been exposed to violence either once (*n* = 21) or repeatedly (*n* = 26). Eighteen participants have only received psychological assistance, one only pharmacological treatment, three both types of assistance, and thirteen no treatment. Moreover, 80.8% of the participants (*n* = 38) manifested PTSD. Global score Mean = 28.89 ± 12.56 (cut point 20).

Regarding the BDI-II and BAI-II instruments, 25.5% of the participants (*n* = 12) displayed severe depression (Mean = 21.72 ± 8.69), while 57.4% (*n* = 27) severe anxiety symptoms (Mean = 26.63 ± 10.68).

**Figure 1:**
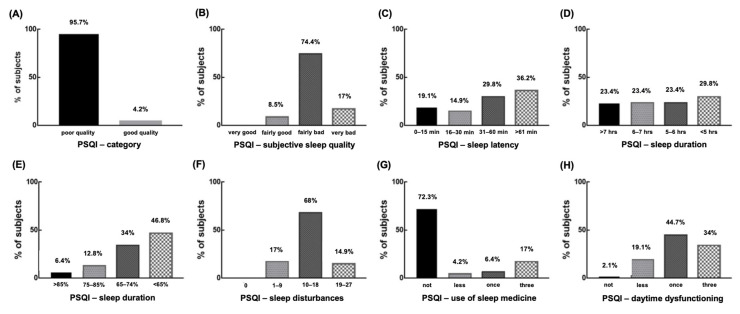
Frequency distribution for the Pittsburgh Sleep and Quality Index (PSQI), category and components. (**A**) PSQI category. Subjects with score > 5 were classified as having poor sleep quality. (**B**–**H**) show PSQI components. (**B**) Subjective sleep quality. (**C**) Sleep latency. (**D**) Sleep duration. (**E**) Sleep efficiency. (**F**) Sleep disturbances. (**G**) Use of sleep medicine. (**H**) Daytime dysfunctioning.

The PSQI values revealed that 95.7% of the participants (*n* = 45) had poor sleep quality (mean = 12.14 ± 3.5) and 74.4% (*n* = 35) subjectively perceived sleep quality as bad. Concerning sleep latency, 36.2% (*n* = 17) of the participants take more than 60 min to fall asleep (Figure 1C), while 29.8% (*n* = 14) indicated that they obtain less than 5 h of sleep per night (Figure 1D). Sleep efficiency refers to the percentage of total time in bed actually spent in sleep; good sleep efficiency is considered to be >85%. Only three participants had a good efficiency (6.4%), whereas half of them (46.8%, *n* = 22) presented a sleep efficiency <65% (Figure 1E). A total of 68% (*n* = 32) of the participants reported a high frequency of sleep disturbances (Figure 1F), 72.3% (*n* = 34) indicated not using sleep medication during the past month (see Figure 1G), 44.7% (*n* = 21) manifested daytime dysfunctions once a week, and 34% (n = 16) indicated dysfunctions three times per week (Figure 1H). Sleep disturbances reported by the participants included sleep fragmentation (*n* = 14), sleep restriction (*n* = 18), nightmares (*n* = 9), periodic limb movement syndrome (*n* = 14), obstructive sleep apnea syndrome (*n* = 19), late insomnia (*n* = 21), and insomnia (*n* = 33).

According to the SHPS evaluation, 59.5% (*n* = 28) of the participants manifested regular sleep hygiene practices (mean = 80.40 ± 15.93).

Concerning the ERQ, scores (ranging from 1 to 7) for Cognitive Reappraisal (*M* = 4.71, *SD* = 1.15) and Expressive Suppression (*M* = 3.55, *SD* = 1.56) were similar to those previously reported in general Mexican samples (Olalde, 2016).

Values for the IRI (ranging from 7 to 35 for each factor) indicated that the higher empathic attitudes were related to EC (*M* = 29.42, *SD* = 3.69), followed by PT (*M* = 25.61, *SD* = 4.23), and similar scores for FS (*M* = 21.91, *SD* = 4.84) and PD (*M* = 21.06, *SD* = 5.40). Only scores for PD presented significantly high values when compared with previously reported Mexican samples (*t* (46) = 3.63, *p* = 0.001) (Ahuatzin et al., 2019).

Correlational analyses for values about mental health issues indicated positive correlations between PTSD and depression (*r*^2^ (47) = 0.83, *p* < 0.001) and anxiety (*r*^2^ (47) = 0.75, *p* = 0.001), and between depression and anxiety (*r*^2^ (47) = 0.66, *p* < 0.001). A negative correlation was identified between sleep quality and sleep hygiene practices (*r*^2^ (47) = −0.47, *p* = 0.001). Positive correlations were identified between sleep quality and PTSD (*r*^2^ (47) = 0.58, *p* = 0.001), depression (*r*^2^ (47) = 0.59, *p* < 0.001), and anxiety (*r*^2^ (47) = 0.51, *p* < 0.001). Negative correlations were observed between sleep hygiene practices and PTSD (*r*^2^ (47) = −0.39, *p* = 0.006) and anxiety (*r*^2^ (47) = 0.31, *p* = 0.03).

Regarding emotional regulation, Cognitive Reappraisal was positively correlated with PTSD severity (*r*^2^ (47) = 0.33, *p* = 0.021) and anxiety (*r*^2^ (47) = 0.37, *p* = 0.009), while Expressive Suppression was positively correlated with PTSD severity (*r*^2^ (47) = 0.36, *p* = 0.012) and depression (*r*^2^ (47) = 0.35, *p* = 0.01). No correlation was found between emotional regulation and sleep quality or sleep hygiene practices.

Concerning empathic attitudes, Personal Distress was positively correlated with PTSD (*r*^2^ (47) = 0.37, *p* = 0.001), depression (*r*^2^ (47) = 0.38, *p* = 0.007), and anxiety (*r*^2^ (47) = 0.31, *p* = 0.03), and negatively correlated with sleep hygiene practices (*r*^2^ (47) = −0.30, *p* = 0.03). No correlation was observed between empathic attitudes and sleep quality.

Most of the participants (80.8%) manifested severe PTSD according to the global severity index. This result contrasts with those of other studies which found that 33.9% of journalist suffered from severe PTSD when covering violent news in Mexico, but not necessarily experiencing direct or indirect attacks or threatening situations causing unemployment and/or family separation (Flores Morales et al., 2014), as it was reported by our participants. Furthermore, the results contrast with observations that 2.3% of women and 0.5% of men in general Mexican samples displayed PTSD after they were exposed to violent situations, according to the National Survey in Psychiatric Epidemiology (Medina-Mora et al., 2005).

In agreement with reports on journalists working in drug-related-violence contexts (Feinstein, 2012), and those on victims of social and political violence in Mexico and other countries (Flores Martínez & Atuesta, 2018), the participants in this study also exhibited depression and anxiety. Although the severity of PTSD was positively correlated with both depression and anxiety, the latter may be more prevalent in our sample, since it relates to the persecution and life-threating events experienced by our participants. Also, PTSD symptoms are associated with stress reactions involving cortisol release and consequent sympathetic nervous functions characterizing states of anxiety (Hori & Kim, 2019).

Exposure to violence may also affect sleep quality (Gallegos et al., 2019). This connection could explain the poor sleep quality and sleep disturbances exhibited by most of the participants. Positive correlations between poor sleep quality and PTSD, depression, and anxiety may suggest comorbid symptoms, as previously reported (Miller et al., 2017).

It should be noted that 59.5% and 29.7% of the participants manifested regular and good sleep hygiene practices, respectively, suggesting that the poor sleep quality observed in 95.7% of the participants is not necessarily due to the lack of sleep hygiene but rather to the presence of sleep disturbances (e.g., insomnia, nightmares) and PTSD, depression, and anxiety symptoms triggering sleep restriction and fragmentation.

Some authors propose that violence and sleep restriction may affect emotional regulation and empathy (Ribero-Marulanda & Novoa-Gómez, 2019; Dorrian et al., 2019). Unexpectedly, our results suggest that poor sleep quality does not affect emotional regulation, as evaluated by the ERQ. Nevertheless, Cognitive Reappraisal was correlated with anxiety, while Expressive Suppression was correlated with depression, suggesting that different emotional regulation strategies may be used and strengthened to deal with depression and/or anxiety symptoms in therapeutic interventions for people who have experienced some form of violence.

Concerning empathic attitudes, Personal Distress values were higher in the participants of this study than in Mexican populations that have not been exposed to violence. These values were positively correlated with PTSD, depression, and anxiety symptoms, and negatively correlated with sleep hygiene practices, suggesting that Personal Distress results in extremely sensible attitudes towards the suffering of others, which may trigger affective disorders and hinder self-care practices favoring good sleep (Gordon et al., 2017). Therefore, assessments and therapeutic interventions should be attuned to individuals who may encounter distress when recalling traumatic experiences.

## 4. Study 2: Neuropsychological and Polysomnographic Evaluation

### 4.1. Participants and Procedure

Twelve participants who took part in Study 1 opted to continue their involvement in Study 2 (Mean age = 38.33 ± 11.9), comprising seven women (mean age = 37.77 ± 13.33, min = 22, max = 56 years old) and five men (mean age = 39.16 ± 10.76, min = 33, max = 60) (see Table 3).

During the initial session, each participant underwent evaluation using two neuropsychological instruments (Neuropsi; BANFE-2) to assess cognitive functions, including memory, language, attention, perception, and decision-making abilities.

In the subsequent session, two experts in psychiatry and sleep medicine conducted clinical interviews based on the International Classification of Sleep Disorders, the MINI-International Neuropsychiatric Interview, and the Toronto Alexithymia Scale.

During the third and final session, participants underwent overnight polysomnography, and self-administered questionnaires were completed to assess parameters related to their perception of sleep, such as sleep latency and subjective awakenings, before and after the study. 

### 4.2. Instruments

The Neuropsychological Battery of Executive Functions and Frontal Lobes (BANFE-2) (Flores-Lázaro et al., 2016) includes a series of tests aimed at evaluating executive functions primarily dependent on the prefrontal cortex, with an approximate administration time of 50 min.

The MINI-International Neuropsychiatric Interview (MINI, Spanish version 5.0.0) is a structured diagnostic interview exploring the presence of symptoms of major neuropsychiatric disorders listed in Axis I of the DSM-5, with an average administration time of 45 min.

The Toronto Alexithymia Scale (Martínez-Sánchez, 1996) assesses the presence of alexithymia, with an approximate administration time of 15 min.

Regarding the Polysomnographic Recording and Sleep Perception, at 20:00 h, the participant was prepared for an all-night (8 h) polysomnographic (PSG) study with a Cadwell Easy 2, version 2 instrument (Cadwell Industries Inc., Kennewick, WA, USA) employing the 10–20 international standard setup to detect electroencephalographic activity, electrooculogram, electromyogram, electro-cardiogram, and nasobucal airflow; thoracic–abdominal movement, oxygen saturation, and body position were also recorded.

### 4.3. Results and Discussion

Several participants exhibited neurocognitive impairments, particularly in memory functions (both encoding and retrieval), attention, concentration, and language. Evaluation with the BANFE-2 revealed that most of the participants demonstrated normal (*n* = 9) and high–normal (*n* = 4) performance in global assessment. Only one participant exhibited mild–moderate impairment, while another showed severe impairment. Specifically, high–normal (*n* = 4) and normal (*n* = 9) performances, and mild–moderate (*n* = 1) and severe impairments (*n* = 1), were observed for functions associated with the dorsolateral cortex, such as planning, working memory, verbal and design fluency, and complex problem-solving, mental flexibility, hypothesis generation, work strategies, seriation, and sequencing. However, mild–moderate impairment (*n* = 4), normal (*n* = 6), and high–normal performances (*n* = 4) were observed in functions linked to the ventromedial cortex, which include attention, inhibition, and conflict detection and resolution. Functions associated with the orbitofrontal cortex, related to emotion processing and regulation, exhibited mild–moderate impairment in some participants (*n* = 4), while others showed normal performance (*n* = 8) (details in Table 7, pretreatment data).

Previous research has indicated that individuals exposed to traumatic events often exhibit cognitive deficits, particularly in memory and attention (Djelantik et al., 2020). Additionally, hypoactivity in the frontal cortex and reduced connectivity with brain regions involved in fear expression have been observed in trauma survivors. Our findings suggest that traumatic experiences may impact orbitofrontal and ventromedial prefrontal regions associated with emotion regulation, attention, conflict detection and resolution, and behavioral inhibition.

Six participants demonstrated difficulties in identifying and describing emotions, as assessed by the Toronto Alexithymia Scale. Interestingly, these individuals also displayed impairments in functions dependent on the orbitofrontal and ventromedial cortices, suggesting a link between emotional regulation difficulties and neurocognitive deficits.

Psychiatric interviews identified various disorders among participants, with depression being the most prevalent (*n* = 12), followed by post-traumatic stress disorder (PTSD) (*n* = 9) and generalized anxiety disorder (*n* = 9). Other disorders, including dysthymic disorder (*n* = 5), panic disorder (*n* = 4), alcohol dependence (*n* = 2), social phobia (*n*= 2), obsessive–compulsive disorder (*n* = 1), and hypomania (*n* = 1), were also observed, though less frequently (see Table 3). These findings align with previous studies indicating a high comorbidity between PTSD and other psychiatric disorders, notably depression and anxiety (Pandi-Perumal & Kramer, 2010; Shrivastava et al., 2014).

In terms of sleep evaluation, participants reported a wide range of bedtime hours, between 23:00 and 4:00 h. with waking times also varying significantly, between 6:00 and 14:00 h. Most individuals (*n* = 10) experienced a total sleep time (TST) below reference values, suggesting sleep restriction among the majority of evaluated individuals.

These results underscore the complex interplay between neurocognitive impairments, psychiatric comorbidities, emotional regulation difficulties, and sleep disturbances among trauma survivors, highlighting the importance of comprehensive assessment and tailored interventions in clinical practice.

**Figure 2:**
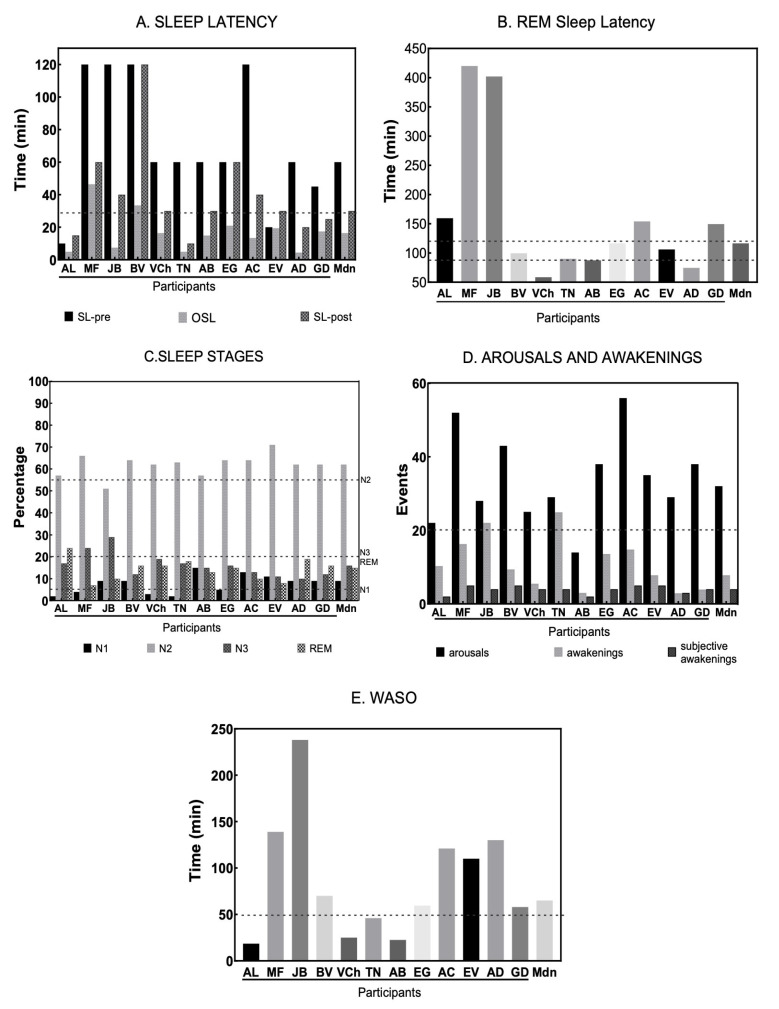
Results for the polysomnographic study (PSG). (**A**) Sleep Latency: SL-pre = subjective sleep latency manifested by the participants before the PSG study describing their regular sleep; OSL = Objective Sleep Latency measured by the PSG study; SL-post = Subjective Sleep Latency manifested by the participants the morning after the PSG study. (**B**) REM Sleep Latency: Rapid Eye Movement Sleep Latency measured by the PSG study. (**C**) Sleep Stages: Percentage of total sleep time concerning the distributions of N1, N2, N3 and REM sleep stages as measured by the PSG study. (**D**) Arousal and awakenings: index of arousal (number of micro-awakenings per hour); total of 1 min. awakenings during all-night measured during the PSG study; subjective awakenings reported by the participants the morning after the PSG study. (**E**) Wake After Sleep Onset: periods of wakefulness after defined sleep onset.

The median objective sleep latency (SL) was 16.25 min, differing from both pre-study subjective SL (median = 60 min) and post-study subjective SL (median = 30 min). However, significant variability in sleep latency was observed among participants, ranging from 4.5 to 46.5 min. While most participants showed values within the reference range (*n* = 8), four individuals had shorter sleep latencies, and three had longer latencies compared to reference values. A shortened sleep latency may be associated with sleep restriction, the effects of antidepressant medications, or obstructive sleep apnea disorder, while a long sleep latency is related to insomnia or anxiety disorders and is an indicator of poor sleep quality (Gupta, 2013) (see Figure 2).

For rapid eye movement (REM) sleep latency, the median value (116.5 min) fell within the reference range (90–120 min). Nevertheless, considerable variability was observed among individuals. In most cases (*n* = 7), values exceeded normal ranges, whereas three participants had REM latencies below adult reference levels, and five participants had expected latencies. Changes in REM sleep latency are biological markers for a wide range of sleep disorders. Specifically, a REM sleep latency of 40 to 70 min has been correlated with psychiatric illnesses such as depression, while its increase is observed in patients with sleep disorders like periodic limb movements, obstructive sleep apnea, and insomnia (Gupta, 2013).

The percentage of time spent in sleep stages N1 (median = 9%) and N2 (median = 62%) exceeded the expected values for healthy adults (5% and 55%, respectively), whereas percentages for N3 (median = 16%) and REM (median = 15%) were below reference values (20% and 20%, respectively). Considerable variability was also observed in this parameter among individuals. The amount and percentage of N1 sleep stage is an indicator of sleep fragmentation, with a high percentage in N1 indicating continuous awakenings that may be caused by sleep disorders such as sleep apnea, periodic limb movements, snoring, bruxism, and insomnia. The N3 stage is considered deep sleep, or slow-wave sleep; a decrease in the percentage of this stage was observed in most cases. The reduction in N3 stage, considered deep sleep, can occur due to the effects of certain medications such as benzodiazepines and barbiturates, insomnia, or mental disorders such as depression, anxiety, post-traumatic stress disorder, and alcoholism (Gupta, 2013).

The median sleep efficiency (SE) was 79%, lower than the expected value according to reference parameters (85%). Only five participants exhibited sleep efficiency within the expected range, with two of them having values very close to, but below, this threshold, while the majority (N = 8) had lower sleep efficiency than expected for healthy adults. Scores for the number of subjective awakenings per participant (median = 4) were similar to reference values; however, the number of objective awakenings was higher (Mdn = 31) than expected in all participants, suggesting an underestimated perception of awakenings. Additionally, the microarousal index per hour (Mdn = 7.85) fell within the expected score for a healthy adult population; nevertheless, there was also high variability, with five individuals showing indices higher than expected. Regarding wakefulness after sleep onset (WASO), the majority of individuals (n = 10) exhibited high scores compared to the reference value (see Table 4).

Moreover, the study identified sleep disorders: primary snoring only (*n* = 2), bruxism only (*n* = 3), periodic limb movements (*n* = 3), and obstructive sleep apnea (*n* = 1). Additionally, several participants presented more than one sleep disorder: primary snoring and bruxism (*n* = 3), obstructive sleep apnea and bruxism (*n* = 3). One individual exhibited obstructive sleep apnea and bruxism, along with abnormal electroencephalographic activity characterized by isolated bursts of slow wave angular waves in the left frontotemporal region. While this finding may be associated with issues in language articulation and production, further correlation with clinical data in a neurological evaluation is warranted. Therefore, observation was recommended, as the individual did not express any impairment (see Figure 2, Table 4)

Regarding wakefulness after sleep onset (WASO), the majority of individuals (n = 10) exhibited high scores compared to the reference value, which has been linked to signs of anxiety and depression (Gupta, 2013) (see Table 4).

The results of Study 2 indicate that following traumatic events, 87% of individuals experience disruptions in sleep patterns, as previously observed by other authors (Maher et al., 2006). Notably, parasomnias, insomnia, and nightmares were prevalent, although periodic limb movement disorder and respiratory disorders like obstructive sleep apnea were also reported, leading to sleep cycle fragmentation and alterations in sleep patterns (Germain, 2013; Blom et al., 2016).

## 5. Study 3: Therapeutic Intervention

### 5.1. Participants and Procedure

The 12 individuals who participated in Study 2 progressed to the subsequent stage, Study 3.

One week following the neuropsychological and neuropsychiatric evaluations, as well as the sleep study, an intervention based on Cognitive Behavioral Therapy (CBT) for sleep disorders commenced. This intervention included two group sessions and four individual sessions, each lasting one hour. The interventions were conducted by two expert therapists (A. M.M. and D. G-R), both with several years of clinical experience, trained in Cognitive Behavioral Therapy (CBT) for sleep disorders and sleep medicine, and members of our research team.

Each group consisted of five participants. The individual sessions followed the Cognitive Behavioral Therapy for Insomnia (CBT-I) manual developed by Michael Perlis (Perlis et al., 2005). For further details about the specific sections of the therapy, please also refer to the article by Rossman (Rossman, 2019). Nevertheless, the following paragraphs provide a general overview of the therapeutic procedure. It is worth noting that the components and strategies of the intervention were tailored slightly for each participant, depending on the specific sleep disorder(s) they exhibited.

(1) The initial group session focused on psychoeducation regarding sleep habits. At the conclusion of this session, the therapist instructed the group on how to maintain a sleep diary. Additionally, individual sessions were arranged with each participant.

(2) Over the subsequent four weeks, CBT sessions for sleep disorders were conducted individually, targeting the specific disorder(s) identified for each patient. Based on this, the therapist selected strategies to work with each patient according to the following components: sleep consolidation (also known as sleep restriction), stimulus control, cognitive restructuring, sleep hygiene, relaxation techniques. For patients with nightmare disorder, the therapist conducted Imagery Rehearsal Therapy (Krakow & Zadra, 2010).

(3) Starting from Session 1, and in each subsequent session, participants were asked to subjectively rate their improvement in sleep quality on a scale from “0 to 10”. They were also asked to assess their mood and functionality in relation to their quality of life. The sleep diary was then reviewed in detail, and the difficulties expressed by the participant in improving their sleep hygiene were identified. The therapist would then focus on instructing the patient in CBT strategies, such as relaxation, cognitive restructuring, stimulus control, etc. To close the session, the therapist gave instructions on activities, tools or homework to practice or readjust (again depending on the sleep disorder). Finally, feedback from the patient about the session was requested.

During each CBT session, the progress of each participant was qualitatively monitored and evaluated through free self-reports and quantitatively through sleep diary parameters, including total time in bed (TTB), total sleep time (TST), sleep latency, subjective wake-up after sleep onset (WASO), number of awakenings, sleep efficiency (SE), and subjective sleep rating. The recordings were reviewed weekly throughout the four-week intervention period (see Table 5).

**Figure 3:**
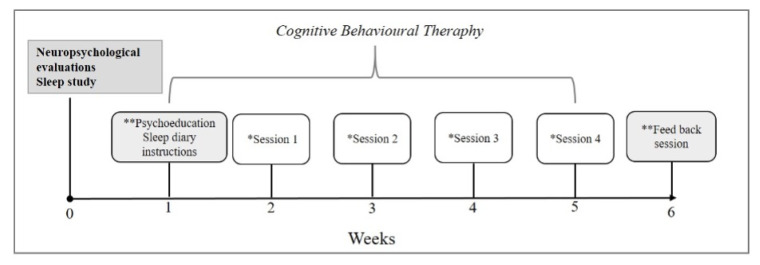
Procedure of CBT for the treatment of sleep disorders. ** Group sessions; * individual sessions.

One week following the conclusion of the CBT program, participants from each group convened for a feedback session (as outlined in Figure 3), during which they were reassessed regarding the effectiveness of CBT on sleep disorders, cognitive function, and mental health.

Due to the health contingency, and as a preventive measure against COVID-19 transmission, the treatment was conducted through online sessions using video call software and virtual meetings via “Zoom: video conferencing”. Several previous reports have indicated that both in-person and digital therapy are equally effective and yield long-term effects (Luik et al., 2020; Luik et al., 2019; Ohayon, 2006). The treatment was conducted in groups: one group comprised six individuals, and two groups comprised three individuals each.

### 5.2. Assessment During Intervention

Throughout the intervention, seven components of the sleep diary were consistently monitored, with most being reviewed at the commencement of each session with every participant to estimate their progress. These components or parameters included total time in bed (TTB), total sleep time (TST), sleep latency (SL), wake time after sleep onset (WASO), awakenings, sleep efficiency (SE), and sleep rating.

To effectively address most sleep disorders, it is imperative for patients to express concerns regarding both the quality and quantity of their sleep, as well as any impact on their work, social interactions, and mood performance over the preceding four weeks. Consequently, it was crucial to elicit feedback from participants regarding their perceived sleep quality and its impact on their overall quality of life upon the conclusion of therapy sessions. During the follow-up session, participants were asked to rate, on a scale from 0 to 5, the extent of improvement in their quality of life: 0 = worsened, 1 = not improved, 2 = the same, 3 = slightly improved, 4 = considerably improved, 5 = fully improved. Table 6 below presents the results of these evaluations for three participants, illustrating the explanation that some patients gave for their reported score and how input from both the therapist and the companion was integrated. Testimonials from participants highlight some of the effects of CBT across the sessions, particularly shedding light on improvements in sleep and their subsequent impacts.

### 5.3. Assessment After Intervention

Upon the completion of the Cognitive Behavioral Therapy sessions, participants received a booklet containing the following self-administered questionnaires and psychometric scales, mirroring those utilized in the diagnostic phase of Study 1 and the psychometric evaluation of Study 2: The Sleep Hygiene Questionnaire (EPHS); Pittsburgh Sleep Quality Index (CPCS); Beck Depression Inventory (BDI-II); Beck Anxiety Inventory (BAI-II); Spanish-Validated Posttraumatic Stress Scale (EGSR); and the Toronto Alexithymia Scale. This approach facilitated the assessment of therapy’s impact on participants’ mental health status, sleep hygiene, and sleep quality, as well as their ability to recognize and articulate their own and others’ emotions.

Following the completion and return of the self-administered instruments assessing mental health and sleep quality and hygiene, each participant attended an individual session to evaluate their cognitive functions, utilizing the two neuropsychological batteries employed in Study 2, Neuropsi and BANFE-2. Data from one participant was excluded from the analysis due to discontinuation of their participation.

### 5.4. Results and Discussion

Total time in bed (TTB) showed a general decrease over the sessions, with noticeable inter-individual variability, but increased during the last week (see Table 4). Excessive time spent in bed is frequently reported among individuals with insomnia, mainly due to poor sleep hygiene (Kryger et al., 2017). Conversely, total sleep time (TST) increased across the sessions for most individuals, indicating adherence to treatment guidelines. However, a few individuals experienced a reduction or no change in TST during the final week, suggesting difficulties in adhering to treatment associated with personal issues, relationship problems, work stress, or recent distressing events.

Sleep latency (SL) decreased consistently across the sessions for all individuals, with scores falling within the normal reference range (between 10 and 20 min) by the last week. Wake time after sleep onset (WASO) decreased progressively over the sessions, and despite inter-individual variability, all individuals remained within the normal reference range (1 to 20% of total sleep time) (Qureshi et al., 2011; Gupta, 2013) (see Table 5).

The number of awakenings decreased as the sessions progressed, with all participants falling within the reference parameters (<5 awakenings per night) (Qureshi et al., 2011; Gupta, 2013). Sleep efficiency increased over the sessions for the majority of participants (*n* = 10), reaching reference parameters (>85%) (Qureshi et al., 2011; Gupta, 2013) (see Table 5).

Improvement in sleep hygiene practices was observed in most participants after the CBT sessions: scores indicating regular sleep hygiene (*n* = 8) or poor sleep hygiene (*n* = 3) improved to good sleep quality (*n* = 8) and excellent sleep hygiene (*n* = 3) (see Table 7).

Following the therapeutic intervention, the Pittsburgh Sleep Quality Index showed lower scores in most individuals, interpreted as an improvement in sleep quality (see Table 7)

Notably, after the CBT sessions, symptoms of depression (*n* = 9) and anxiety (*n* = 11) improved in most participants, as indicated by the results of the BDI-II and BAI-II, respectively. Symptoms of post-traumatic stress disorder, present in all participants before CBT treatment, also decreased in most of them (n = 10), both in the global score and in most of the subscales, except for “hyperactivation” (see Table 7).

Regarding the Toronto Alexithymia Scale, results indicate that, before and after treatment, only one participant exhibited scores indicating difficulty in identifying and communicating their own and others’ feelings.

Table 8 displays the Mean ± S.D. of the normalized scores divided according to the cortical region evaluated in the executive functioning of the participants. Before the intervention, most participants showed normal (*n* = 8) and high–normal (*n* = 4) performance in the global evaluation of executive functions. The majority (*n* = 10) exhibited normal performance in the functioning of the dorsolateral cortex, related to processes such as planning, working memory, verbal and design fluency, complex problem-solving, mental flexibility, hypothesis generation, work strategies, seriation, and sequencing. Regarding the functioning of the orbitofrontal cortex (primarily responsible for emotion regulation and processing, and behavioral control) and the ventromedial cortex (related to processes of inhibition, conflict detection and resolution, as well as attention), two individuals showed mild to moderate impairment.

After the CBT sessions, an improvement in the performance of executive functions was observed in the majority of evaluated individuals. In fact, no cases of mild to moderate impairment were observed, with distributions in normal and high–normal levels (see Table 8).

## 6. General Discussion

The current study highlights a pronounced prevalence of affective disorders concomitant with sleep disturbances and emotional dispositions among journalists, human rights defenders, and relatives of disappeared persons who have undergone severe violence and are seeking governmental support.

Our investigation demonstrates that individuals subjected to violence, irrespective of its frequency or nature, manifest a distinct psychological, psychiatric, and cognitive profile. There exists a notable comorbidity between neuropsychiatric disorders, notably depression, generalized anxiety disorder, post-traumatic stress disorder (PTSD), and dysthymic disorder, alongside other conditions such as agoraphobia, psychoactive substance abuse, social phobia, and hypomania, though to a lesser extent (Pandi-Perumal & Kramer, 2010; Shrivastava et al., 2014).

A majority of participants displayed alterations in sleep latency, both at sleep onset and during rapid eye movement (REM) sleep. These findings suggest a potential association with insomnia or sleep fragmentation. Furthermore, observations indicate that most individuals experienced superficial sleep, characterized by a higher percentage of N1 and N2 sleep stages, along with reduced percentages of N3 and REM sleep stages, multiple awakenings, sleep efficiencies below 85%, and sleep durations below the recommended norms for the adult population (Gupta, 2013; Qureshi et al., 2011).

Regarding cognitive functions, most participants exhibited deficits in attention, concentration, memory encoding and retrieval, as well as executive functions associated with emotion regulation, behavioral control, and conflict resolution. Meta-analyses suggest that a substantial proportion of individuals (50 to 70%) exposed to traumatic events experience cognitive impairment post-trauma, primarily affecting memory, attention, and learning cognitive functions (Qureshi et al., 2011; Schuitevoerder et al., 2013; Martindale et al., 2017).

Several authors have encouraged integrating sleep disorder management into the clinical treatment of neuropsychiatric disorders, particularly PTSD. Neglecting sleep disorders may exacerbate symptoms, hindering physical and cognitive restoration during nocturnal sleep (Germain, 2013; Koren et al., 2002; Maher et al., 2006; Nappi et al., 2011; Drummond et al., 2006; Krystal, 2012). Chronic sleep restriction has been linked to judgment errors, impulsivity, impaired threat recognition, empathy deficits, emotional dysregulation, and cognitive function alterations (Koren et al., 2002; Maher et al., 2006; Nappi et al., 2011; Drummond et al., 2006; Germain, 2013). Hence, the deficits observed in participants may be attributed in part to disruptions in sleep patterns and sleep disorders leading to insomnia, multiple awakenings, sleep fragmentation, and non-restorative sleep.

Given the delicate psychosocial and institutional circumstances of our participants and the presence of sleep disorders, we proposed a Cognitive Behavioral Therapy (CBT) intervention for sleep, anticipating that its effects would extend to comorbid symptomatology such as depression, anxiety, and post-traumatic stress (Manber & Carney, 2015; Wickwire, 2019).

The CBT intervention was delivered online via video conference sessions. Previous studies have demonstrated the effectiveness of both in-person and virtual therapy, with sustained effects over the long term (Luik et al., 2020; Luik et al., 2019; Ohayon, 2006; Werner-Seidler et al., 2018; Germain et al., 2012). Our results indicate that virtual CBT improved sleep quality and continuity, as assessed through parameters reported in the sleep diary and supported by testimonials and explanations provided by participants, therapists, and therapeutic companions.

The therapy primarily employed tools for addressing insomnia, nightmares, circadian rhythm disturbances, scheduling regulation strategies, and relaxation techniques, all of which were identified sleep issues among participants. Following therapeutic intervention, a reduction in the severity of anxiety, depression, and post-traumatic stress disorders was observed in the majority. These findings are consistent with prior research indicating that sleep-focused Cognitive Behavioral Therapy (CBT) ameliorates symptoms associated with depression, anxiety, and post-traumatic stress, potentially mitigating feelings of fear and distress during sleep (Maher et al., 2006; Manber & Carney, 2015; Wickwire, 2019; Ramsawh et al., 2016; Taylor et al., 2018; Carletto et al., 2017).

In terms of assessing cognitive functions, research indicates that sleep fragmentation contributes to cognitive deficits in individuals with post-traumatic stress disorder (Drummond et al., 2006; Schuitevoerder et al., 2013; Lipinska et al., 2014; Girardeau & Lopes-dos-Santos, 2021). Our study demonstrates that following the therapeutic intervention, individuals achieved normal to above-normal cognitive performance scores, suggesting that improving sleep quality enhances cognitive function. Extensive evidence supports the role of sleep in the brain dynamics essential for long-term memory consolidation (Dudai et al., 2015; Hamel et al., 2023; Ramos & López, 2010).

While our study provides valuable insights, it also has limitations that should be considered with caution. Larger samples are necessary to validate our results considering the effect sizes present in psychological categories (Funder & Ozer, 2019), as well as explore potential differences across diverse participant categories (e.g., journalists, human rights defenders, relatives of disappeared persons), and to explore gender differences and psychoactive substance use, with careful consideration of additional variables influencing mental health. Moreover, our study was contextualized in the so-called Mexican “war on drugs,” which started in 2006, but data from the National Survey in Psychiatric Epidemiology was published just before this context (Medina-Mora et al., 2005); updated data are necessary to contrast our results with those obtained from the general population. Another relevant point pertains to the characteristics of the sample. All participants joined the study voluntarily, demonstrating high levels of motivation and receiving social support from governmental institutions or civil society organizations. Given the influence of these factors on the positive outcomes of Cognitive Behavioral Therapy (Berntsen & Christensen, 2013), they should be carefully considered in future research.

Despite these limitations, our work contributes to designing and implementing sensitive evaluations to identify mental health issues in individuals affected by violence seeking government assistance. The prevalence of PTSD, depression, and anxiety underscores the urgent need for support. Given the precarious state of the mental health system in Mexico and substantial delays in accessing care (Ramos & López, 2010), academic institutions could collaborate with government entities to aid victims of violence. Table 9 summarizes key findings, strengths, and limitations of our study, as well as and future directions.

Violent experiences profoundly impact various facets of an individual, including their personality, mental health, cognition, and sleep quality. Addressing one affected element may facilitate the restoration of others (Koren et al., 2002; Maher et al., 2006; Nappi et al., 2011; Drummond et al., 2006; Germain, 2013). Evaluation of sleep quality, disturbances, and hygiene practices can be enhanced through non-invasive polysomnographic methods, serving as primary variables for identifying mental health concerns. Furthermore, interventions for poor sleep and disturbances, promise not only for improving sleep quality but also for addressing other psychiatric symptoms and disorders, such as nightmares, depression, and anxiety related to PTSD (Harvey et al., 2003). Leveraging emotional regulation strategies and empathic attitudes displayed by participants can facilitate the implementation of finer, more cooperative interventions.

## Figures and Tables

**Table 1:** Strengths and limitations associated with the referenced studies.

Strengths	Limitations
Comprehensive documentation of the psychological impact of violence, including PTSD, depression, and anxiety, supported by statistics from credible national institutions.	Lack of longitudinal studies to explore the long-term effects of violence-related trauma and sleep disturbances.
Emphasis on the interplay between sleep disturbances, cognitive decline, and emotional regulation, offering a holistic understanding of trauma’s impact on individuals.	Limited availability of descriptive-controlled studies addressing mental health interventions in high-risk populations, such as journalists and human rights defenders.
Identification of Cognitive Behavioral Therapy (CBT) as a first-line intervention for sleep disorders in PTSD, consistent with international guidelines.	Minimal research on specific cultural and contextual factors affecting emotional regulation and sleep quality in trauma-exposed populations.
Clear focus on the urgent need for mental health interventions, especially for populations subjected to repeated victimization and social stigma.	Over-reliance on cross-sectional data rather than experimental or interventional studies to establish causal relationships.
Highlights the scarcity of mental health and sleep-focused interventions in governmental programs, underscoring gaps in current support systems.	Limited exploration of gender-specific and occupational differences in the psychological and cognitive impacts of violence.

**Table 2:** General data of the participants.

Data	*n*
**Gender**	
Women	29
Men	18
**Category**	
Journalists	6
Human rights defender	28
Relatives of disappeared person	8
Victim of assault	5
**Education**	
Master’s degree	8
Bachelor’s degree	28
Unfinished bachelor’s degree	4
High school	2
Junior high school	4
Elementary school	1
**Civil status**	
Divorced	3
Married	4
In union	6
Single	34
**Birthplace**	
Mexico City (MEX)	30
State of Mexico (MEX)	4
Guerrero (MEX)	3
Sinaloa (MEX)	2
Guadalajara (MEX)	2
Michoacán (MEX)	1
Chiapas (MEX)	1
United States of America	1
Honduras	1
Bolivia	1
Argentina	1

**Table 3:** General data for the participants in Study 2, including gender and age; condition associated with violence, type of violence, and frequency of violence experienced; neuropsychiatric disorders and sleep disorders identified by the MINI-International Neuropsychiatric Interview, and sleep disorders as identified by a clinical interview based on International Classification of Sleep Disorders.

I.D.	Gender/Age	Condition Associated with Violence, Type of Violence, and Frequency of Violence Experienced	Neuropsychiatric Disorders	Sleep Disorders
A.L.	Female 27 y.o.	Activist (Human Rights Defender) Direct Once	Hypomaniac Disorder Panic Disorder PTSD	Insomnia Primary snore
M.F.	Female 42 y.o.	Activist (Human Rights Defender) Both Types Repeated	Major Depression Panic Disorder PTSD Gen. Anxiety Disorder Dysthymic Disorder Agoraphobia	Insomnia PLMS mild Bruxism Nightmares
J.B.	Male 41 y.o.	Journalist Direct Repeated	Major Depression PTSD	Circadian rhythm disorder, delayed phase PLMS severe OSA Nightmares
B.V.	Female 45 y.o.	Journalist Direct Repeated	Major Depression Panic Disorder PTSD Gen. Anxiety Disorder Social Phobia	Insomnia PLMS mild
V.Ch.	Male 41 y.o.	Activist (Human Rights Defender) Indirect Once	Major Depression Gen. Anxiety Disorder	Insomnia
T. N.	Male 41 y.o.	Activist (Human Rights Defender) Direct Repeated	Major Depression PTSD	Primary snore Nightmares
A.B.	Male 33 y.o.	Activist (Human Rights Defender) Indirect Repeated	Major Depression PTSD Gen. Anxiety Disorder	Insomnia
E.G.	Female 25 y.o.	Activist (Human Rights Defender) Indirect Repeated	Major Depression Gen. Anxiety Disorder Dysthymic Disorder	Insomnia OSA
A.C.	Male 34 y.o.	Activist (Human Rights Defender) Both Types Repeated	PTSD Gen. Anxiety Disorder	Insomnia
E.V.	Female 36 y.o.	Relatives of Disappeared Persons Indirect Once	Major Depression Panic Disorder PTSD Alcohol Dependence	Insomnia OSA Nightmares
A.D.	Female 21 y.o.	Activist (Human Rights Defender) Both Types Once	Major Depression Gen. Anxiety Disorder	Insomnia OSA
G.D.	Female 27 y.o.	Relatives of Disappeared Persons Indirect Once	Major Depression Panic Disorder Dysthymic Disorder	Insomnia OSA Nightmares

**Table 4:** Sleep pattern parameters during PSG for Study 2.

Parameters	*M* ± S.E.	Median (IQR)	Reference Values
TST	378.2 ± 134.02	392.4 (108.1)	420–540
SL (min)	61.6 ± 40.51	60 (35.3)	10–20
REM SL (min)	156.5 ± 111.7	116.5 (69.97)	90–120
WASO (min)	156.5 ± 111.7	70 (59.8)	20% of TTS
N1 (%)	8 ± 0.42	9 (7.25)	5
N2 (%)	63 ± 0.70	62 (5.7)	55
N3 (%)	16 ± 0.06	16 (6.5)	20
REM (%)	14 ± 0.05	15 (7.5)	20
SE (%)	78 ± 0.13	79 (18.98)	>85
PLMS (# per hour)	9.8 ± 7.14	7.8 (5.8)	<10
Total awake (# total)	11.2 ± 7.3	9.85 (11.63)	<5

Note. TST = total sleep time, SL = sleep latency, REM SL = REM sleep latency, WASO = wake after sleep onset, REM = rapid eye movement, SE = sleep efficiency, PLMS = periodic limb movements.

**Table 5:** Median parameters of sleep diary evaluated during each session of Cognitive Behavioral Therapy over four weeks.

Weeks	TTB (Hours) Mdn, (IQR)	TST (Hours) Mdn (IQR)	SL (min) Mdn	WASO (% from TST) Mdn (IQR)	SE (%) Mdn (IQR)	Total Awakings Mdn (IQR)	Sleep Satisfaction (0–10 Subjective Scale) Mdn, (IQR)
PT	7.75 (2)	6.75 (1.12)	60 (63.75)	30 (25.22)	75 (9.7)	2 (1)	7 (1.25)
1	8.74 (1.35)	6.71 (1.81)	63.48 (50.25)	10.06 (18.29)	78.05 (13.47)	1.44 (1.41)	7.11 (1.47)
2	8.68 (1.71)	6.71 (1.35)	37.1 (39.28)	6.43 (15.57)	81.21 (20.37)	1.60 (1.52)	7.50 (1.71)
3	7.50 (1.88)	6.41 (1.43)	21.67 (20.35)	8.75 (13.37)	86.02 (7.1)	1.09 (1.62)	7.71 (1.29)
4	8.33 (1.74)	7.02 (1.09)	20.83 (11.25)	8.21 (12.21)	89.31 (8.61)	0.79 (1.77)	8.1 (1.15)

Note. Mdn = median, IQR = (interquartile range), TTB = total time in bed, TST = total sleep time, SL = sleep latency, WASO = wake after sleep onset, SE = sleep efficiency.

**Table 6:** Examples of the participants and the therapist testimonials regarding their improvement and behavior during the intervention.

I. D.	Sleep Disorder(s)	Treatment Approach	Rating of Patient Improvement Degree (0–5)	Therapist Observation
T.N.	Insomnia, nightmares	Schedule restructuring therapy, sleep hygiene, imagery rehearsal theraphy for nightmares	“I rate the level of improvement as a 4, because I think this is a process I need to work on. I feel improvement in my mood and concentration, I even resumed reading. I feel like I still need to work on the time I spend in bed and completely leaving my phone before sleep. It helped me a lot to become aware of my sleep behaviors.”	“She showed a lot of willingness from the beginning of the therapy. She adhered to the therapy‘s rules and agreements, although she was always realistic about what she believed she could or could not do. She only had nightmares for one week, stemming from a threatening message. However, she received treatment that same week and adhered properly, which led to the disappearance of the nightmares. She was progressing well in improving her sleep quality, except for the last week when the presence of menstrual cramps significantly affected her well-being.”
J.B.	Circadian rhythm disorder, delayed phase PLMS, OSA	Sleep hygiene, cronotherapy	“I rate my level of improvement as a 4.5, I just need this pandemic situation to end to be at 100%. I noticed a complete change from the person I was before. Now I sleep at normal people‘s hours, not like a vampire. I no longer fall asleep in the early hours and wake up early, I open the windows to ventilate and expose myself to daylight, something I never did before, I used to live with artificial light. Even my acquaintances tell me that I am a different person, because I completely changed my routine.”	“From the outset, I noticed him as a willing and participative individual. During the evaluations and throughout the polysomnographic recording, he was very engaged and interested in the entire process. However, he did not attend the psycho-education session 2 because he fell asleep, and throughout session 1, he was yawning and not paying attention. During the initial sessions with the therapist, he mentioned arriving late. However, throughout sessions, improvements were observed along with greater adherence to the treatment; he appeared willing, attentive, with a more active demeanor and better mood. He showed awareness that the treatment is gradual and requires his commitment.”
B.V.	Insomnia, nightmares, PLMS	Schedule restructuring therapy. Therapy for nightmare treatment. Cognitive Behavioral Therapy for insomnia.	“The rating I would give for improvement is 4. I have noticed an improvement in my skin; I no longer spend much time in bed, only the necessary time to sleep. My concentration and memory have improved; for example, I now remember the names of the characters in the series I watch (laughs). I hardly eat sweets anymore, and the amount of cola drink consumption has also decreased; I only have a small can (300 mL) in the afternoon, before 6 PM. Also, I now only sleep with one pillow; before, I used to sleep with two because I felt like someone might enter the house. But I feel that there are still some things I need to work on, like depression; sometimes it overwhelms me, and it takes me a while to get up, although those days are rare now. I also have to find a stable place to stay because I haven’t found the right roommate yet, but I’m sure I can work that out.”	“She was very willing and cooperative, and she expressed gratitude for the treatment. She is very sensitive and cries easily. At the beginning of therapy, her face had a sad and flat expression. However, I noticed a clear change at the end of therapy; she was capable and determined to make decisions to improve her quality of life, as she expressed to me herself.”

**Table 7:** Questionnaires, indexes and scales for mental health and sleep hygiene and sleep quality evaluated pre- and post-CBT for sleep disorder treatments.

Questionnaires, Index, Scales and Subscales	Pre-Mdn (IQR)	Post-Mdn (IQR)	Level of Severity or Range by Scale
PTSD Symptom Severity Scale. Overall score Re-experiencing Avoidance Hyper-arousal Cognitive alteration and negative mood	29.5 (17.25) 5.5 (6) 5 (4.5) 11 (3.5) 10 (5.5)	19.5 (12.25) 4 (3.5) 2.5 (1.75) 7 (5.75) 5 (7.5)	Overall score, cut point = 20 Re-experiencing, cut point = 4 Avoidance, cut point = 4 Hyper-arousal, cut point = 6 Cognitive alteration, cut point = 6
Beck Depression Inventory	22 (14.25)	11 (11.75)	Minimum = 0–13 Mild = 14–19 Moderate = 20–28 Severe = 29–63
Beck Anxiety Inventory	29 (16.5)	15 (12.25)	Minimum = 0–7 Mild = 8–15 Moderate = 16–25 Severe = 26–63
Sleep Hygiene Questionnaire	67.5 (17.75)	112.5 (27.05)	Poor sleep hygiene = 0–30 Bad sleep hygiene = 31–60 Regular sleep hygiene = 61–90 Good sleep hygiene = 91–120 Excellent sleep hygiene = 121–150
Pittsburgh Sleep and Quality Index (PSQI)	14.5 (3.5)	5 (2.75)	Cut point = 5

Note. Mdn = Median; IQR = (Interquartile range).

**Table 8:** Normalized scores of executive function performance evaluated with the Neuropsychological Battery of Executive Functions and Frontal Lobes for pre-treatment (PRE) and post-treatment (POST) stages.

		Participants												
Scores		A.L	M.F.	J.B.	B.V.	V.Ch	T.N.	A.B.	E.G.	A.C.	E.V.	A.D.	G.D.	*M* ± S.D.
**Orbitofrontal**	**Pre**	94	76	79	115	104	91	113	109	109	106	73	104	104 ± 14.85
	**Post**	110	125	103	106	91	106	110	125	123	118	-	104	110 ± 10.72
**Ventromedial**	**Pre**	131	97	118	97	79	97	95	124	124	104	82	74	97± 18.84
	**Post**	85	90	118	90	131	90	95	126	125	118	-	101	101 ± 17.40
**Dorsolateral**	**Pre**	122	109	108	118	97	98	106	124	122	98	67	88	107 ± 16.59
	**Post**	103	113	106	122	130	113	113	113	106	120	-	96	113 ± 9.48
**Overall**	**Pre**	122	102	104	121	96	96	107	124	125	100	64	88	103 ± 17.75
	**Post**	102	113	108	122	130	113	108	125	124	124		98	113 ± 10.48

**Table 9:** Summary of key findings, strengths, limitations, and future directions of the present study.

Key Findings	Strengths	Limitations	Future Directions
High prevalence of PTSD (80.8%), anxiety (57.4%), and depression (25.5%) in participants. 95.7% of participants with poor sleep quality, frequent insomnia and nightmares. Cognitive deficits in attention, memory, and decision-making, correlated with poor sleep and PTSD. CBT-based intervention improved sleep quality, reduced anxiety, depression, and PTSD symptoms. Emotional regulation strategies and empathic attitudes, correlated with mental health outcomes.	Multiphase design incorporating psychometric, neuropsychological, and polysomnographic assessments. Comprehensive evaluation of sleep disturbances using validated tools. Detailed neurocognitive evaluations identified specific domains of impairment. Tailored CBT intervention for sleep disorders addressing individual needs. Integration of emotional and cognitive measures to understand trauma’s broader impacts.	Small sample size (n = 47 for initial study, n = 12 for detailed assessments), limiting generalizability. Limited ability to disentangle effects of specific sleep disorders from comorbid psychiatric conditions. Lack of a control group prevents definitive causal inferences. Relatively short intervention period; follow-up effects are unclear. Limited exploration of cultural and contextual factors influencing emotional regulation.	Expand sample size and include diverse populations to validate findings and explore subgroup differences. Investigate longitudinal impacts of improved sleep quality on mental health outcomes. Conduct randomized controlled trials to confirm efficacy of interventions. Evaluate long-term sustainability of CBT outcomes and explore digital CBT modalities. Study cultural adaptation of CBT interventions in diverse settings.

## Data Availability

Data is contained within the article.
